# Substrate-Mediated Regulation of Src Expression Drives Osteoclastogenesis Divergence

**DOI:** 10.3390/genes15091217

**Published:** 2024-09-18

**Authors:** Bo Hu, Yiming Chen, Yuman Li, Chenyu Deng, Yuting Niu, Zhewen Hu, Yao Li, Shiyu Sun, Ying Huang, Xuliang Deng, Yan Wei

**Affiliations:** 1 Department of Geriatric Dentistry, Peking University School and Hospital of Stomatology & National Center for Stomatology & National Clinical Research Center for Oral Diseases & National Engineering Research Center of Oral Biomaterials and Digital Medical Devices, Beijing 100081, China; klluobu@163.com (B.H.); chenym@pku.edu.cn (Y.C.); kqliyuman@bjmu.edu.cn (Y.L.); niuyt698@163.com (Y.N.); philip0323@163.com (Z.H.); liyao2018@126.com (Y.L.); hying031129@163.com (Y.H.); kqdengxuliang@bjmu.edu.cn (X.D.); 2 Department of Prosthodontics, Peking University School and Hospital of Stomatology & National Center for Stomatology & National Clinical Research Center for Oral Diseases & National Engineering Research Center of Oral Biomaterials and Digital Medical Devices, Beijing 100081, China; 3 Department of Orthodontics, Peking University School and Hospital of Stomatology & National Center for Stomatology & National Clinical Research Center for Oral Diseases & National Engineering Research Center of Oral Biomaterials and Digital Medical Devices, Beijing 100081, China; dengchenyu@pku.edu.cn; 4 Department of General Dentistry, Peking University School and Hospital of Stomatology & National Center for Stomatology & National Clinical Research Center for Oral Diseases & National Engineering Research Center of Oral Biomaterials and Digital Medical Devices, Beijing 100081, China; shiyu_yeo@sina.com

**Keywords:** osteoclastogenesis, substrate, Src, cytoskeletal remodeling, oxidative phosphorylation (OXPHOS)

## Abstract

Background/Objectives: Glass, bone, and dentin are commonly applied substrates for osteoclast cultures; however, the impact of these substrates on osteoclastogenesis remains underexplored. This study aimed to address a significant gap in understanding how different substrates influence the process of osteoclastogenesis. Methods: RAW 264.7 cells were cultured and induced with RANKL on glass, bone, and dentin slides. Histological and molecular techniques were used to identify patterns and differences in osteoclast behavior on each substrate. Results: Osteoclasts cultured on glass slides possessed the greatest number of nuclei and the highest expression levels of ACP5 (TRAP) and CTSK, with osteoclasts on bone and dentin slides displaying progressively lower levels. Src expression was also most pronounced in osteoclasts on glass slides, with decreased levels observed on bone and dentin. This variation in Src expression likely contributed to differences in cytoskeletal remodeling and oxidative phosphorylation (OXPHOS), resulting in substrate-dependent divergences in osteoclastogenesis. Conclusions: Glass slides were the most favorable substrate for inducing osteoclastogenesis, while bone and dentin slides were less effective. The substrate-induced expression of Src played a fundamental role in shaping the phenotypic divergence of osteoclasts. These insights fill important knowledge gaps and have significant implications for the development and selection of in vitro models for bone-related diseases and drug screening platforms.

## 1. Introduction

Osteoclasts are indispensable in the physiological and pathological remodeling of bone tissues. Consequently, elucidating the regulatory mechanisms of osteoclastogenesis is a critical aspect of biomedical research and clinical therapy [1,2,3,4]. The attachment of osteoclasts to a culture substrate is a fundamental step in their subsequent differentiation and functionalization [5,6]. While glass, bone, and dentin slides are all frequently used substrates in osteoclast cultures, there remains a knowledge gap regarding how or whether these different substrates influence the process of osteoclastogenesis [7,8].

At present, there is tremendous controversy over which type of substrate is most suitable for inducing osteoclastogenesis. Specifically, bone and dentin slides, both of which are derived from natural mineralized tissue, preserve critical characteristics of in vivo microenvironments. The morphology of bone and dentin slices is consistent with that of mineralized tissue in vivo [9,10,11]. In addition, the typical structures found in the extracellular matrix, including the amino acid sequence RGD, are preserved on bone and dentin slides [12,13]. Therefore, these slides are regarded as favorable substrates for inducing osteoclastogenesis. However, recent studies have also indicated that substrate stiffness is a crucial factor contributing to osteoclastogenesis, making glass slides superior to bone and dentin due to their higher stiffness [9]. Moreover, the divergence in expression levels of functional hallmarks such as ACP5 (TRAP), RANK, and CTSK, which are regarded as prominent indicators of resorption behavior, in osteoclasts cultured on different substrates remains to be examined [14]. Consequently, studies on osteoclastogenesis may obtain heterogeneous results due to the various choices of culture substrates [15,16]. Thoroughly examining the differences in osteoclast behavior across substrates and elucidating their underlying mechanisms could provide significant insights into the field, potentially leading to more effective approaches in both research and clinical practice.

The present study offers the first comprehensive comparison of the biological features of osteoclasts cultured on bone slides, dentin slides, and glass slides. We determined that osteoclasts cultured on glass slides possessed the greatest number of nuclei along with the highest expression of functional markers, followed by osteoclasts cultured on bone and then on dentin slides. Using Smart-Seq and immunofluorescence assays, we found that the divergent expression of Src on different substrates is the fundamental factor leading to variance in osteoclastogenesis. Expression of Src subsequently enhanced cytoskeletal remodeling and oxidative phosphorylation (OXPHOS), which ultimately facilitated the functionalization of osteoclasts. Overall, the above results elucidate the mechanisms of how the culture substrate affects osteoclastogenesis. Our findings may also provide a critical reference for studies on osteoclasts when choosing a culture substrate.

## 2. Materials and Methods

### 2.1. Preparation of Culture Substrates of Osteoclasts

We employed three different culture substrates as a model system for osteoclast differentiation: cell culture glass slices (Solarbio, Beijing, China), bovine cortical bone slices (IDS, Bolton, UK), and human dentin slices. The dentin slices for the cell culture were obtained from human premolars and molars that were extracted for medical purposes. These slices were sectioned using a low-speed diamond saw with water cooling, a procedure approved by the Ethics Committee of Peking University School of Stomatology (PKUSSIRB-2024100091). Subsequently, all substrates were subjected to ultrasonic cleaning in distilled water, sterilized, and stored in phosphate-buffered saline at 4 °C until required for the experimental assays.

### 2.2. Cell Culture and Osteoclast Differentiation

The RAW 264.7 macrophage lineage, sourced from PH Biotechnology (Wuxi, China), was cultured at 37 °C in a humidified atmosphere containing 5% CO_2_. The culture medium was DMEM (Procell, Wuhan, China) enriched with Earle’s salts, L-glutamine, NaHCO_3_, 10% fetal bovine serum (FBS; Corning Inc., Corning, NY, USA), and 1% penicillin–streptomycin (Procell, Wuhan, China). In order to initiate osteoclast differentiation, cells were placed on glass, bone, or dentin slides in 48-well plates (Corning Inc., Corning, NY, USA) at a density of 7500 cells/cm^2^. To induce osteoclastogenesis and observe the differentiation process, α-MEM (Procell, Wuhan, China) enriched with 100 ng/mL of a soluble receptor activator of the NF-κB ligand (RANKL; Amizona Scientific, Birmingham, AL, USA) was applied from Day 2 to Day 5 [17,18]. Four days was determined to be the most optimal duration for RANKL-induced osteoclast differentiation on the glass, bone, and dentin slices. The culture medium was refreshed every other day throughout the experiments. 

### 2.3. Tartrate Resistant Acid Phosphatase (TRAP) Staining and Quantitative Analysis

Osteoclastogenesis was evaluated by performing assays to identify TRAP-positive multinucleated cells (more than three nuclei) using a Tartrate Resistant Acid Phosphatase (TRAP) Staining Kit from Amizona Scientific (Birmingham, AL, USA). 

The assay employed the naphthol-AS-MX phosphate method. In this process, cells are treated with a solution that includes 0.05 mol/L acetate buffer (pH 5.0), 0.27 mmol/L naphthol AS-MX phosphate, 1% N,N-dimethylformamide, 1.6 mmol/L Fast Red LB salt, and 50 mmol/L sodium tartrate. This solution is maintained at 37 °C, which allows the TRAP enzyme to react with the naphthol substrate, producing a red precipitate in TRAP-positive cells. Following the reaction, the cells are rinsed and examined under a microscope to identify TRAP-positive multinucleated cells characterized by their red staining and more than three nuclei, indicating successful osteoclast differentiation [18].

TRAP-positive areas were quantified using ImageJ software (version Java 1.8.0_345; National Institutes of Health, Bethesda, MD, USA). The statistical analysis was conducted using a one-way ANOVA test (GraphPad Prism, version 10.0; GraphPad Software, San Diego, CA, USA) to evaluate the significance across different groups. A *p*-value of less than 0.05 was considered statistically significant.

### 2.4. Immunofluorescence and Confocal Laser Scanning Microscopy

On Day 4 of RANKL stimulation, osteoclasts cultured on glass, bone, and dentin substrates were fixed with 4% paraformaldehyde for 10 min, washed three times with PBS, infiltrated with 0.1% Triton X-100 for 10 min, and blocked with 3% bovine serum albumin (BSA) for 1 h at room temperature. After being washed three times with PBS, osteoclasts on different substrates were incubated with the primary antibodies overnight at 4 °C, followed by incubation with the secondary antibodies for 1 h. After staining the cell nuclei and f-actin ring with DAPI (Solarbio, Beijing, China) and Rhodamine-labeled phalloidin (Solarbio, Beijing, China), the samples were sealed with 50% glycerol. Fluorescence pictures were captured using a confocal microscope (Leica, TCS-SP8 STED 3X, Wetzlar, Germany). Quantitative analysis of the fluorescence signals was conducted using the Leica Application Suite X (LAS X) software (LAS_X_4.7.0). 

The antibodies used were as follows: c-SRC (Proteintech, Wuhan, China), ATP6V0D1 (Proteintech, Wuhan, China), COXIV (Proteintech, Wuhan, China), CD9 (Proteintech, Wuhan, China), SYN1 (Proteintech, Wuhan, China), DC-STAMP (Bioss, Beijing, China), Tartrate Resistant Acid Phosphatase (Abcam, Cambridge, UK), anti-rabbit IgG-Alexa Fluor^®^ 488 (Abcam, Cambridge, UK), and anti-mouse IgG-Alexa Fluor^®^ 647 (Abcam, Cambridge, UK).

### 2.5. Scanning Electron Microscopy

Next, we performed scanning electron microscopy (SEM) analysis. After four days of RANKL stimulation, osteoclasts cultured on the glass, bone, and dentin slices were first fixed in 2.5% glutaraldehyde and then dehydrated with a graded series of ethyl alcohol (30%, 50%, 70%, 80%, 90%, 95%, and 100%). Following dehydration, the specimens were coated with a thin layer of gold to enhance their conductivity and then analyzed using a scanning electron microscope (FE-SEM, S-4800, HITACHI, Tokyo, Japan) to observe the morphological characteristics of the osteoclasts on different substrates.

### 2.6. RNA Isolation

Osteoclasts cultured on glass, bone, and dentin slices in 48-well plates were used for RNA collection at various differentiation stages to analyze the gene expression profiles. RNA was isolated at four critical time points: at Day 0, from RAW 264.7 macrophages; at Day 3, from pre-osteoclasts; at Day 4, from mature osteoclasts; and at Day 5, from late-stage osteoclasts, with each time point analyzed in triplicate. The total RNA was extracted using TRIzol reagent (Invitrogen, Carlsbad, CA, USA) according to the manufacturer’s protocol.

### 2.7. Library Preparation and Smart-Seq Sequencing

SMARTer cDNA synthesis starts with picogram amounts of total RNA or single cell/several cells. A modified oligo(dT) primer (the SMART CDS Primer) primes the first-strand synthesis reaction. When SMARTScribeTM Reverse Transcriptase reaches the 5′ end of the mRNA, the enzyme’s terminal transferase activity adds a few additional nucleotides to the 3′ end of the cDNA. The carefully-designed SMARTer Oligonucleotide base-pairs with the non-template nucleotide stretch, creating an extended template to enable SMARTScribe RT continue replicating to the end of the oligonucleotide. The resulting full-length, single-stranded (ss) cDNA contains the complete 5’ end of the mRNA, as well as sequences that are complementary to the SMARTer Oligonucleotide. Amplify sscDNA by LD PCR and get enough dscDNA for library construction.

cDNA was fragmented by dsDNA Fragmentase (NEB, M0348S) by incubate at 37 °C for 30 min. Library construction begins with fragmented cDNA. Blunt-end DNA fragments are generated using a combination of fill-in reactions and exonuclease activity, and size selection is performed with provided sample purification beads. An A-base is then added to the blunt ends of each strand, indexed Y adapters are ligated to the fragments, and the ligated products are amplified with PCR. And then we performed the paired-end sequencing on an Illumina NovaseqTM 6000 at LC Bio Technology CO., Ltd (Hangzhou, China) following the vendor’s recommended protocol. Subsequently, data quality was verified with Fastp software (https://github.com/OpenGene/fastp, accessed on 13 December 2023), and high-quality reads were mapped to the mouse reference genome using HISAT2 (https://daehwankimlab.github.io/hisat2/, accessed on 3 February 2024).

### 2.8. Data Processing and Differential Gene Expression Analyses

After sequencing, the samples were normalized and analyzed with the R package DESeq2. Significantly differentially expressed genes were defined at |Log2FC| ≥ 1 and a *p* value of ≤ 0.05. Gene set enrichment analysis (GESA) enrichment analysis were performed using the R packages cluster profiler and pathview (GO: http://geneontology.org, KEGG: http://www.kegg.jp/kegg, accessed on 1 September 2024). The gene numbers and *p* values of related terms were visualized using the R package ggplot2. Heatmaps, clustering maps, and density plots were drawn with the R package ggplot2.

## 3. Results

**Optimal duration of RANKL-induced osteoclastogenesis on glass, bone, and dentin substrates is 4 days.**

**Figure 1:**
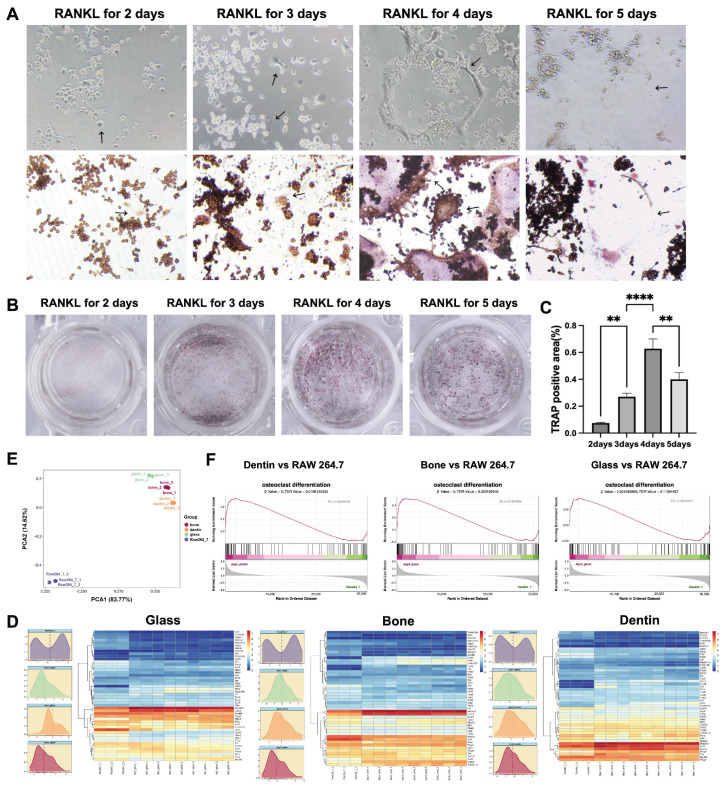
Optimal duration of RANKL-induced osteoclastogenesis on glass, bone, and dentin substrates (4 days). (**A**) Light microscopy and TRAP staining recorded the differentiation of osteoclasts derived from RAW 264.7 macrophages cultured on glass from Day 2 to Day 5. TRAP-positive cells with more than three nuclei were classified as mature osteoclasts. Scale bar = 100 μm. The arrows indicated the process from osteoclastogenesis to apoptosis. (**B**) Macroscopic images of TRAP-stained osteoclasts in a 48-well plate. (**C**) Quantitative analysis showed that TRAP-positive areas peaked on Day 4, with a significant increase from Day 3, followed by a decline on Day 5 (** *p* < 0.01, **** *p* < 0.0001). (**D**) Principal component analysis (PCA) identified distinct variance patterns among osteoclasts on glass, bone, and dentin substrates, as well as RAW 264.7 macrophages. (**E**) Gene Set Enrichment Analysis (GSEA) indicated a significant upregulation of osteoclast differentiation genes across all substrates by Day 4 compared to that of RAW 264.7 macrophages. (**F**) Hierarchical clustering heat maps and temporal density plots illustrated the gene expression profiles associated with the NF-κB signaling pathway in osteoclasts across the three substrates by Day 4.

Various methods are used to differentiate osteoclasts from RAW 264.7 macrophages, resulting in significant variations in the duration required for functionalization [17,19,20,21]. To determine the optimal duration for osteoclast induction, we conducted a sequential induction assay and documented morphological changes during osteoclastogenesis (Figure 1). By Day 4, a significant increase in multinucleated giant cells occurred, followed by signs of apoptosis on Day 5, indicated by cytoskeleton fragmentation (Figure 1A). Meanwhile, TRAP-positive areas peaked on Day 4, followed by a decline (Figure 1B,C).

Concurrently, we performed transcriptomic profiling of osteoclasts cultured on glass, bone, and dentin substrates from Day 3 to 5 using Smart-Seq. Principal component analysis (PCA) and hierarchical clustering revealed significant heterogeneity in gene expression between osteoclasts and RAW 264.7 macrophages (Figure 1D and Figure S1A). Gene Set Enrichment Analysis (GSEA) indicated significant upregulation of osteoclast differentiation markers across all substrates by Day 4 across all substrates (Figure 1E). Temporal analysis of NF-kB signaling pathway genes confirmed that osteoclast differentiation reached its peak by Day 4 on all three substrates, consistent with the TRAP staining results (Figure 1F). Differential gene enrichment analysis detected greater variance between the osteoclasts cultured on glass and the two mineralized slides than between osteoclasts cultured on dentin and bone slides (Figure S1B).

**Enhancement of cell fusion and functional hallmark expression were most significant in osteoclasts cultured on glass substrates, followed by that on bone and dentin substrates.**

**Figure 2:**
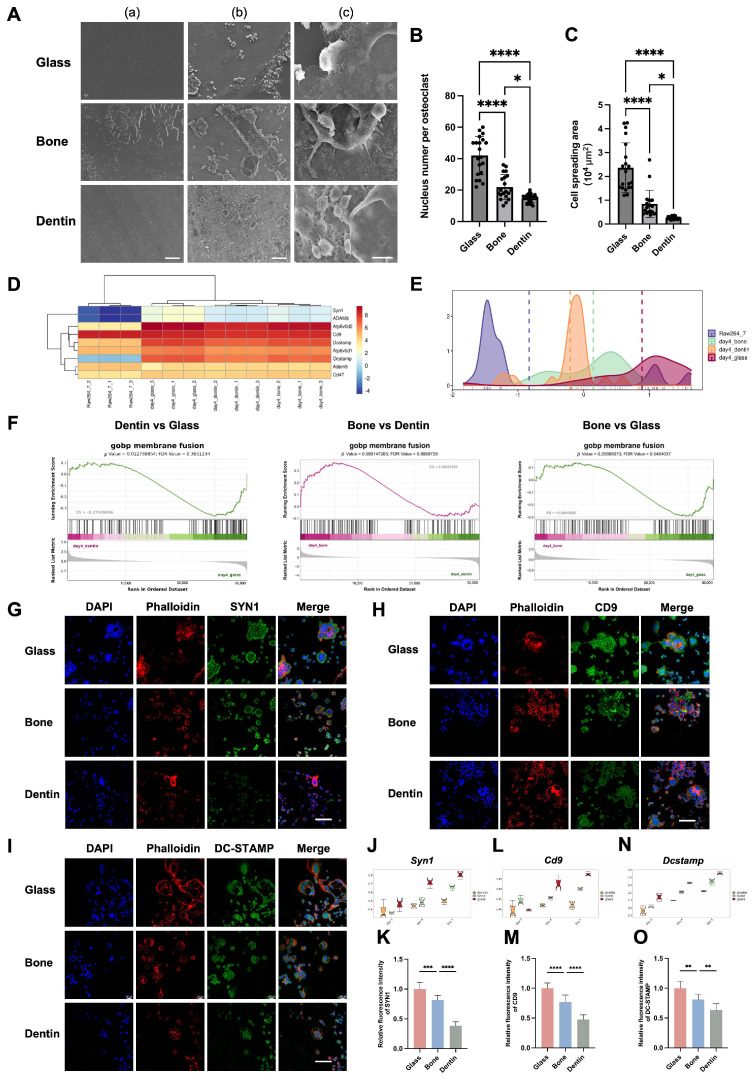
Divergence in fusion activity of osteoclasts cultured on glass, bone, and dentin substrates. (**A**) Scanning electron microscopy (SEM) images highlight pseudopodal formations and cellular interactions of osteoclasts on glass, bone, and dentin substrates. Scale bars = 50 μm (**a**,**b**) and 10 μm (**c**). (**B**) A quantitative analysis of osteoclast spreading area, presented as mean ± SD (* *p* < 0.05, **** *p* < 0.0001). (**C**) Quantitative analysis of nucleus count per osteoclast, also presented as mean ± SD, with the same statistical significance annotations. (**D**) The heatmap of gene expression patterns related to cell fusion in osteoclasts across substrates. (**E**) The density plot indicates the distribution of genes related to cell fusion across different substrates. (**F**) GSEA presents a descending trend in cell membrane fusion gene sets between the glass vs. bone, glass vs. dentin, and bone vs. dentin comparisons. (**G**–**I**) Representative confocal immunofluorescence images displaying SYN1, CD9, and DCSTAMP expression in osteoclasts across substrates. Scale bar = 80 μm. (**J**,**L**,**N**) Quantitative transcriptomic analyses of *Syn1*, *Cd14*, *and Dcstamp* gene expression in osteoclasts cultured on glass, bone, and dentin substrates. (**K**,**M**,**O**) Quantitative analyses of the fluorescence intensity of SYN1, CD9, and DCSTAMP proteins, with data presented as mean ± SD (* *p* < 0.05, ** *p* < 0.01, *** *p* < 0.001, **** *p* < 0.0001).

The fusion of preosteoclasts is a distinctive biological process during osteoclastogenesis, characterized by an increase in both cell size and nuclei number. In this study, scanning electron microscopy (SEM) revealed that osteoclasts on glass substrates were larger and exhibited more extensive pseudopodia and cellular contacts compared to those on bone and dentin (Figure 2A). Furthermore, we observed that osteoclasts cultured on glass slides possessed significantly larger spreading areas and more nuclei, with the smallest size and fewest nuclei observed on dentin (Figure 2B,C and Figure S2A).

Smart-Seq analysis further showed significant upregulation of fusion-related genes in osteoclasts across all substrates, with the highest fusion activity on glass, followed by bone and dentin (Figure 2D–F). Immunofluorescence confirmed that cell fusion markers (SYN1, CD9, DCSTAMP) were most highly expressed on glass slides, with lower levels on bone and dentin (Figure 2G–O).

**Figure 3:**
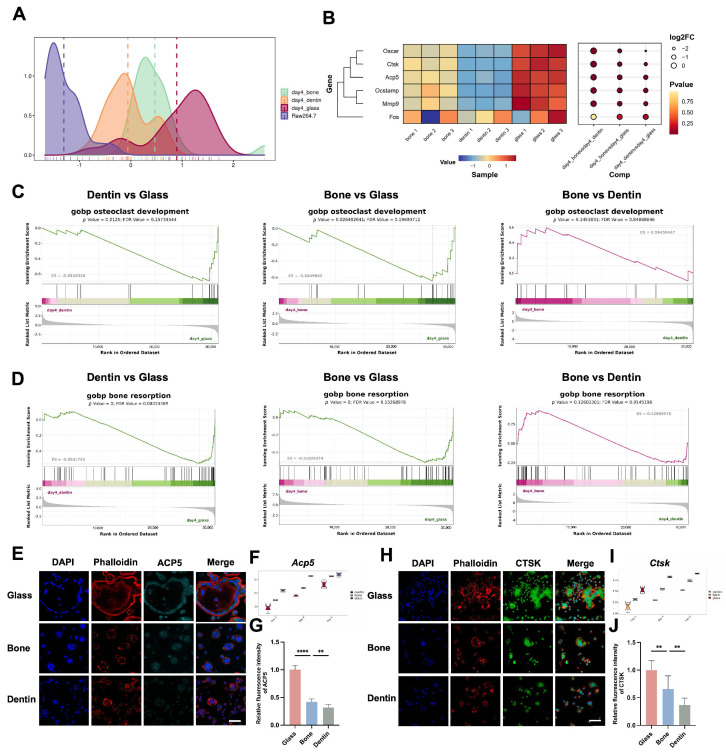
Differential functional hallmark expression in osteoclasts cultured on diverse substrates. (**A**) The heatmap depicts the expression of mature osteoclast hallmarks across glass, bone, and dentin substrates. (**B**) The density plot highlights the distribution of these mature hallmarks for each substrate. (**C**,**D**) The GSEA-GO analysis indicates regulatory trends in gene sets associated with osteoclast development and bone resorption in osteoclasts among the glass vs. bone, glass vs. dentin, and bone vs. dentin groups comparisons. (**E**,**H**) Representative confocal immunofluorescence images of ACP5 (TRAP) and CTSK expression in osteoclasts on the three substrates. Scale bar = 80 μm. (**F**,**I**) Quantitative transcriptomic analyses of *Acp5 (TRAP) and Ctsk* gene expression across these substrates. (**G**,**J**) Quantitative analyses of the fluorescence intensity of ACP5 (TRAP) and CTSK proteins in osteoclasts cultured on these substrates, presented as mean ± SD (** *p* < 0.01, **** *p* < 0.0001).

Apart from cell fusion, elevated functional hallmark expression is also regarded as a typical feature during osteoclastogenesis. In the present study, Smart-Seq revealed upregulation of key functional markers (*Oscar*, *Ctsk*, *Acp5*, *Ocstamp*) across all substrates, with the highest expression on glass, followed by bone and dentin (Figure 3A,B). GSEA-GO analysis highlighted significant differences in osteoclast development and bone resorption, with the highest activity on glass and the lowest on dentin (Figure 3C,D). Immunofluorescence further confirmed the trend of decreasing ACP5 (TRAP) and CTSK expression from glass to bone to dentin (Figure 3E–J).

Overall, our results demonstrate that culture substrates significantly impact cell fusion activity and functional marker expression in osteoclasts, with glass providing the most favorable environment, followed by bone and dentin.

**Expression of Src and subsequent cytoskeletal remodeling account for substrate-induced divergence in osteoclastogenesis.**

**Figure 4:**
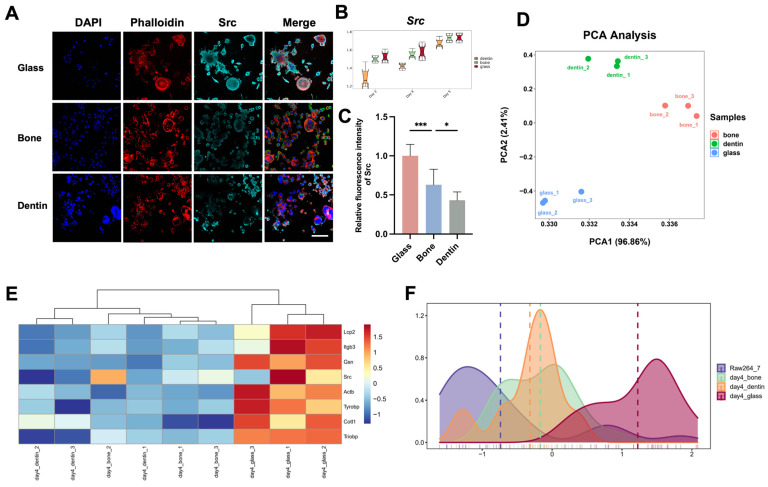
Expression of Src and subsequent cytoskeletal remodeling account for the substrate-induced divergence in osteoclastogenesis. (**A**) Representative confocal immunofluorescence images reveal the expression levels of Src in osteoclasts cultured on glass, bone, and dentin substrates. Scale bar = 80 μm. (**B**) Quantitative transcriptomic analyses of *Src* gene expression in osteoclasts cultured on these substrates. (**C**) Quantitative analyses of the fluorescence intensity of Src proteins in osteoclasts cultured on these substrates, presented as mean ± SD (* *p* < 0.05, *** *p* < 0.001). (**D**) PCA was used to determine the variance patterns in cytoskeletal-related genes among osteoclasts cultured on glass, bone, and dentin substrates. (**E**) Hierarchical clustering heat maps of cytoskeletal gene expression profiles in osteoclasts across the three different substrates on Day 4. (**F**) Density plots present the distribution of genes related to the cytoskeleton in osteoclasts across the three substrates on Day 4.

Apart from variations in osteoclast phenotypes caused by the culture substrate, we explored the underlying mechanisms. It is generally held that osteoclastogenesis is a successive process in which non-receptor tyrosine kinases play a fundamental role [22]. In this study, significant differences in the expression levels of Src, a key member of the tyrosine kinase family, among osteoclasts cultured on different substrates. Specifically, Smart-Seq and immunofluorescence assays demonstrated that Src expression was the highest in osteoclasts cultured on glass slides, with decreasing levels on bone and the lowest levels on dentin (Figure 4A–C). Notably, osteoclasts cultured on glass slides exhibited more diverse morphologies, including asymmetrical and elongated forms, which were associated with higher Src expression.

Previous studies have established that Src plays a key role in regulating cytoskeletal organization and dynamics by modulating the balance between actin filaments and microtubules, which are essential for osteoclast adhesion, migration, and morphological changes, especially during cell spreading and fusion stage.

Our results indicated that Src expression in osteoclasts is substrate-dependent and consistent with fusion activity of osteoclasts on corresponding substrates; we infer that Src may influence cell fusion by regulating the cytoskeleton remodeling. PCA analyses of genes related to cytoskeletal remodeling revealed distinct differences between osteoclasts cultured on different substrates (Figure 4D). Specifically, genes related to cytoskeletal remodeling (e.g., Lcp2, Itgb3, Gsn, Actb, Tyrobp, Cotl1) were most highly expressed on glass, mirroring the trend observed for Src expression (Figure 4E,F).

The above findings highlight the significant impact of culture substrates on Src expression and suggest that substrate-induced variations in Src may influence cytoskeletal remodeling, driving cell fusion during osteolclastogenesis.

**Significant variation in oxidative phosphorylation (OXPHOS) contributes to substrate-driven divergent osteoclast differentiation.**

**Figure 5:**
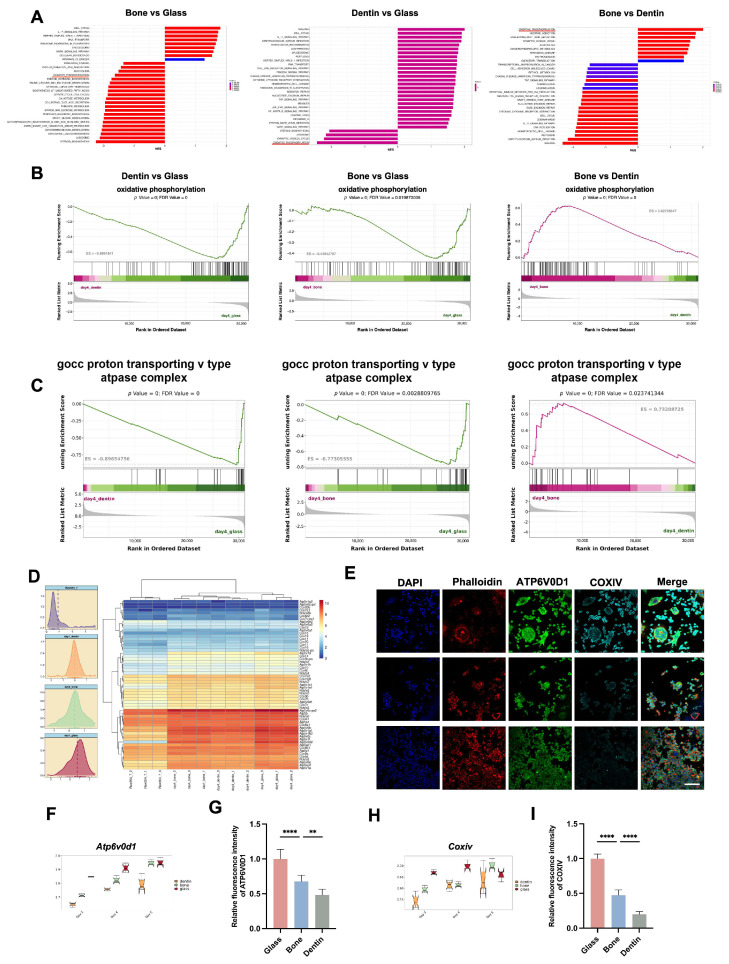
Significant variation in oxidative phosphorylation (OXPHOS) contributes to substrate-induced divergent osteoclast differentiation. (**A**) The GSEA-KEGG analysis indicates that OXPHOS is a significantly enriched pathway across substrate comparisons. (**B**) GSEA-KEGG analysis indicates the presence of regulatory trends in the OXPHSO signaling pathway between the glass vs. bone, glass vs. dentin, and bone vs. dentin comparisons. (**C**) GSEA-GO analysis indicates regulatory trends in proton-transporting V-type ATPase among the glass vs. bone, glass vs. dentin, and bone vs. dentin groups. (**D**) Heatmap and density plots present OXPHOS gene expression profiles in mature osteoclasts cultured on three different substrates and in RAW 264.7 macrophages on Day 4. (**E**) Representative confocal immunofluorescence images show the expression levels of ATP6V0D1 and COXIV in osteoclasts cultured on glass, bone, and dentin substrates. Scale bar = 80 μm. (**F**,**H**) Quantitative transcriptomic analyses of Atp6v0d1 and Coxiv gene expression in osteoclasts cultured on these substrates. (**G**,**I**) Quantitative analyses of the fluorescence intensity of ATP6V0D1 and COXIV proteins in osteoclasts cultured on these substrates, presented as mean ± SD (** *p* < 0.01, **** *p* < 0.0001).

Energy metabolism is essential for osteoclast differentiation [23,24], but the effect of culture substrates on this process remains unclear. In this study, we explored the divergence in energy metabolism among osteoclasts cultured on different substrates. GSEA-KEGG analysis identified oxidative phosphorylation (OXPHOS) as the most significantly enriched term in comparisons between osteoclasts on dentin and glass and between bone and dentin, and it was among the top 20 terms in bone vs. glass (Figure 5A). Moreover, GSEA-KEGG and GSEA-GO analyses indicated a decreasing trend in OXPHOS and proton-transporting V-type ATPase-related gene sets across substrates in the order of glass, bone, and dentin (Figure 5B,C). Variations in OXPHOS-related functions, including mitochondrial activity and ATP synthesis, were also demonstrated (Figure S3A). Notably, despite these differences, the genes related to OXPHOS were significantly upregulated compared to RAW 264.7, suggesting that enhancement of OXPHOS is an important factor contributing to osteoclastogenesis (Figure 5D). Immunofluorescence and quantitative analyses revealed the highest expression levels of ATP6V0D1 and COXIV in osteoclasts on glass, followed by bone and dentin (Figure 5E–I). 

These findings emphasize the substantial impact of culture substrates on osteoclast energy metabolism, particularly OXPHOS. In summary, OXPHOS appears to play a significant role in substrate-induced divergence in osteoclastogenesis, with its activation and ATP synthesis contributing to cell fusion and maturation on different substrates.

**Src may play a central role in correlating the critical biological processes in osteoclastogenesis.**

**Figure 6:**
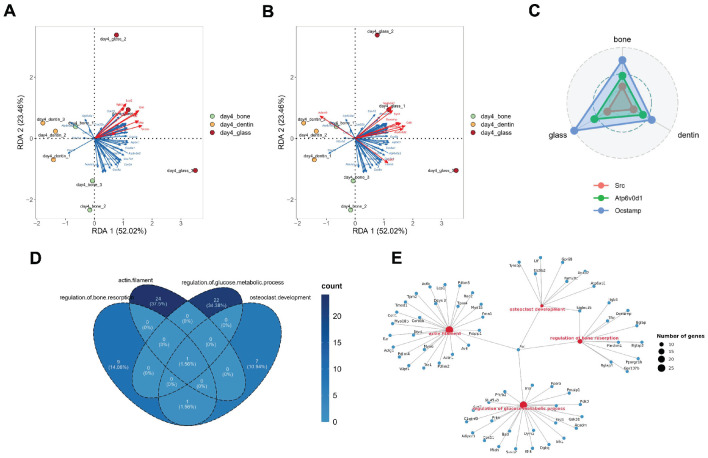
Correlation between genes involved in fundamental biological processes in osteoclastogenesis. (**A**) Redundancy Analysis (RDA) for gene expression related to the cytoskeleton (blue vectors) and oxidative phosphorylation (red vectors) in osteoclasts cultured on glass, bone, and dentin substrates. (**B**) RDA-illustrated gene expression patterns related to OXPHOS (blue vectors) and cell fusion (red vectors) in osteoclasts cultured on glass, bone, and dentin substrates. (**C**) The radar chart displays the comparative expression levels of core genes involved in these crucial biological processes. (**D**) The Venn diagram presents the overlap between genes involved in these biological processes. (**E**) The gene network diagram delineates the regulatory network essential for fundamental biological processes in osteoclastogenesis.

To identify the key factor contributing to the substrate-induced divergence in osteoclastogenesis, we examined the relevance of major biological processes. Redundancy Analysis (RDA) revealed a consistent correlation between cytoskeletal remodeling, OXPHOS, and cell fusion (Figure 6A,B). We also detected identical gene expression patterns of *Src*, *Atp6v0d1*, *and Ocstamp* among the osteoclasts cultured on different substrates, with the highest expression on glass and the lowest on dentin (Figure 6C).

Notably, integrating the interaction networks of genes involved in actin filament organization, osteoclast differentiation, bone resorption regulation, and glucose metabolic pathways, we found that Src serves as a central element connecting all fundamental biological processes (Figure 6D,E).

## 4. Discussion

The culture substrate has a major impact on osteoclastogenesis [9,25]. As a result, studies may draw heterogeneous conclusions due to the selection of different substrates. Hence, the aim of this study was to comprehensively analyze the variance in osteoclasts cultured on different substrates, namely, bone, dentin, and glass slides. We found that the number of nuclei and the expression of functional hallmarks, including ACP5 (TRAP) and CTSK, were highest in osteoclasts cultured on glass slides, followed by bone and dentin. Furthermore, we revealed a relationship between Src expression and osteoclastogenesis, the latter of which was driven by cytoskeletal remodeling and OXPHOS. In general, this study offers the first comprehensive comparison of osteoclasts cultured on bone, dentin, and glass slides. Our findings may improve the understanding of the mechanisms that induce osteoclastogenesis, thereby improving the reproducibility and comparability of research findings on osteoclasts.

Contrary to the conventional understanding that substrates derived from in vivo mineralized tissue are most suitable for inducing osteoclastogenesis, our findings indicate that glass slides are more favorable for osteoclast cultures. Specifically, osteoclasts cultured on glass slides possessed the highest fusion activity and functional marker expression, both of which are commonly used indicators of osteoclast functions [19,26]. Thus, the results indicate that adopting glass slides as culture substrates is an appropriate measure to reduce the difficulty in inducing osteoclastogenesis. Furthermore, we found that osteoclasts cultured on different substrates possessed dramatically divergent cellular features. Specifically, osteoclasts cultured on glass slides were two to three times larger than those cultured on bone or dentin slides. Similar variations were also identified in functional marker expression levels. Therefore, confirming the consistency of culture substrates is crucial when comparing conclusions drawn from different studies.

Beyond the significant differences in cellular features between osteoclasts cultured on different substrates, we also further elucidated the underlying mechanisms. The non-receptor tyrosine kinase family plays a pivotal role in modulating development and disease [27,28,29]. Previous studies stressed the significance of modulating non-receptor tyrosine kinase in osteoclastogenesis regulation [30,31,32]. However, the effect of the culture substrate on non-receptor tyrosine kinase has not yet been explored. In this study, we found that Src, a typical subtype of non-receptor tyrosine kinase involved in various biological processes, is most highly expressed on osteoclasts cultured on glass slides, followed by osteoclasts cultured on bone and dentin slides. Osteoclastogenesis is a complex process consisting of several successive procedures. Consequently, modulating an essential factor induces a cascade of reactions. However, the correlation between the key procedures involved in osteoclastogenesis was not clearly reported in previous studies. In the present study, we found that Src appears to influence the variance in osteoclastogenesis observed on different substrates. Specifically, the expression of Src was positively correlated with cytoskeletal remodeling and OXPHOS, both of which are fundamental processes that drive cell fusion during osteoclastogenesis [33]. This suggests that Src may play an important role in correlating fundamental biological processes in osteoclastogenesis. Moreover, the regulatory relationship between Src and COXIV, as documented in a previous study, further supports this conclusion [34]. Therefore, targeting Src could be a promising strategy for modulating osteoclast activity across different substrates.

The important aspect of interpreting the biological effects observed in this study is the consideration of the physical and chemical properties of the substrates used [9,35]. Glass, with its high stiffness, promotes cytoskeletal tension and cell spreading, enhancing osteoclast fusion and activity [36]. In contrast, the porous structure of dentin, characterized by dentinal tubules, may have an inhibitory effect on osteoclast formation [37]. Interestingly, reports on the impact of surface roughness on osteoclastogenesis are conflicting, with some studies indicating inhibition and others showing enhanced osteoclast formation on rough surfaces [38,39,40]. These variations in substrate properties can influence mechanotransduction pathways in osteoclasts, potentially leading to the observed differences in cell fusion, functional marker expression, and overall osteoclastogenesis.

The findings of this study have significant implications for the development of in vitro models of bone diseases and drug screening platforms. The substrate-dependent divergence in osteoclastogenesis underscores the importance of selecting appropriate culture conditions when modeling bone-related diseases in vitro. The pronounced osteoclastogenesis observed on glass could provide a more sensitive platform for screening potential therapeutic agents targeting osteoclast activity. However, for studies aiming to closely mimic the in vivo bone environment, substrates derived from mineralized tissues, such as bone or dentin, might be more appropriate despite their lower osteoclastogenic potential. This balance between physiological relevance and assay sensitivity is a critical consideration in the design of in vitro models for bone research.

Despite the comprehensive comparison of osteoclasts cultured on different substrates in the present study, some aspects remain to be more fully explored in future research. One limitation of our study is that it could not fully recapitulate the complexity of the in vivo bone microenvironment—a three-dimensional and highly dynamic environment where osteoclasts interact with a complex extracellular matrix (ECM) and are influenced by various mechanical and biochemical cues. Future studies should consider incorporating 3D culture systems to complement the findings obtained from the present study. Additionally, while RAW 264.7 cells are commonly used for osteoclastogenesis studies, bone marrow-derived monocytes (BMMs) provide a closer approximation to in vivo conditions [7,9,19]. Integrating BMMs and 3D culture systems in future research could further elucidate the role of the extracellular microenvironment in osteoclastogenesis and enhance the translational potential of in vitro findings to clinical applications.

## 5. Conclusions

In this study, we noticed that the variable expression of Src on different substrates may play an important role in inducing divergent biological features among osteoclasts. In addition, we demonstrated that glass slides are the most favorable for promoting the fusion activity and expression of functional markers, followed by bone slides and dentin slides. We also found that the elevated expression of Src may mediate cytoskeletal remodeling and OXPHOS, both of which are fundamental processes that drive cell fusion during osteoclastogenesis. Therefore, our study offers significant insights into the biological characteristics of osteoclasts in bone biology and provides a valuable reference for the development and selection of in vitro models for bone research.

## Data Availability

The original contributions presented in the study are included in the article. Further inquiries can be directed to the corresponding author.

## Supplementary Materials

The following supporting information can be downloaded at https://www.mdpi.com/article/10.3390/genes15091217/s1, Figure S1: (A) The heatmap illustrates significant transcriptional heterogeneity among osteoclasts cultured on glass, bone, and dentin substrates compared to RAW 264.7 macrophages. (B) The volcano plots show the number of upregulated (red) and downregulated (blue) genes between the glass vs. bone, glass vs. dentin, and bone vs. dentin comparisons. Figure S2: (A) Representative confocal immunofluorescence images show the spreading area and nucleus of osteoclasts cultured on glass, bone, and dentin substrates. Scale bar = 80 μm. Figure S3: (A) The GSEA-GO analysis uncovers the variation in downstream biological functions related to OXPHOS across osteoclasts cultured on different substrates.
