# Supplementary figures and images for: Substrate-Mediated Regulation of Src Expression Drives Osteoclastogenesis Divergence
Source: Genes (Basel). 2024 Sep 18;15(9):1217. doi: 10.3390/genes15091217 (PMC11431296; doi:10.3390/genes15091217)

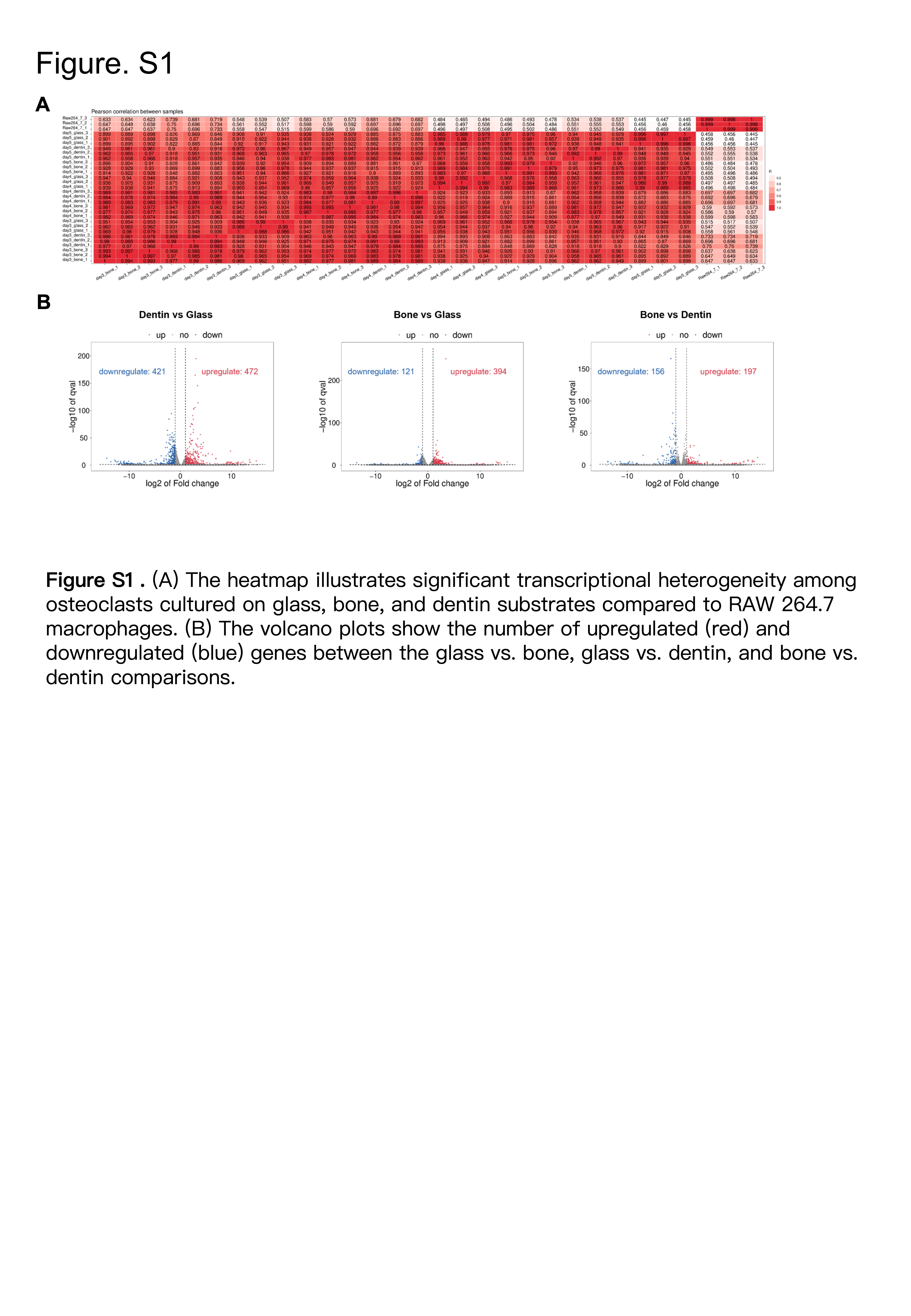

Supplement: Supplementary file 1 [file genes-15-01217-s001.zip › Figure S1.png]

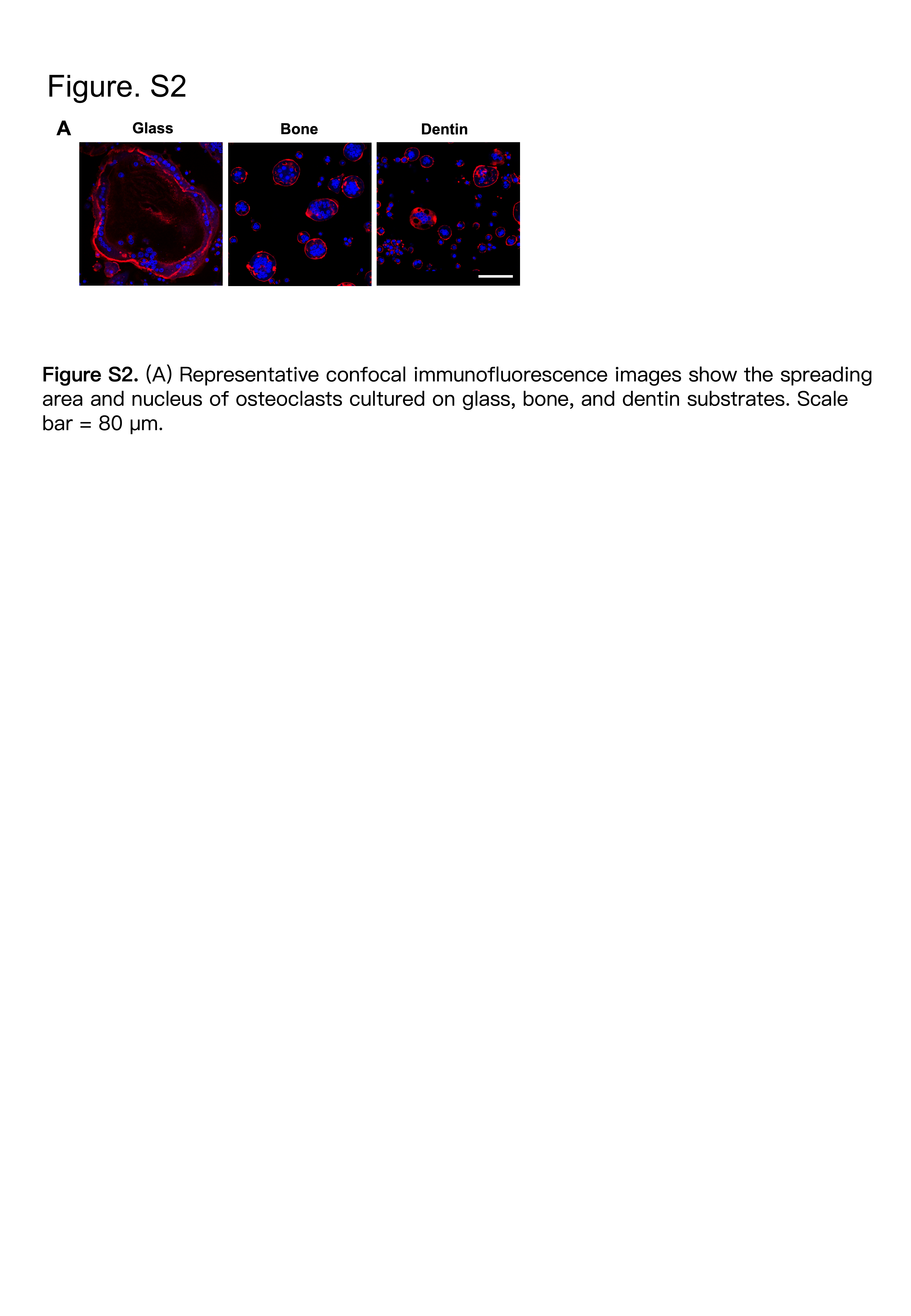

Supplement: Supplementary file 1 [file genes-15-01217-s001.zip › Figure S2.png]

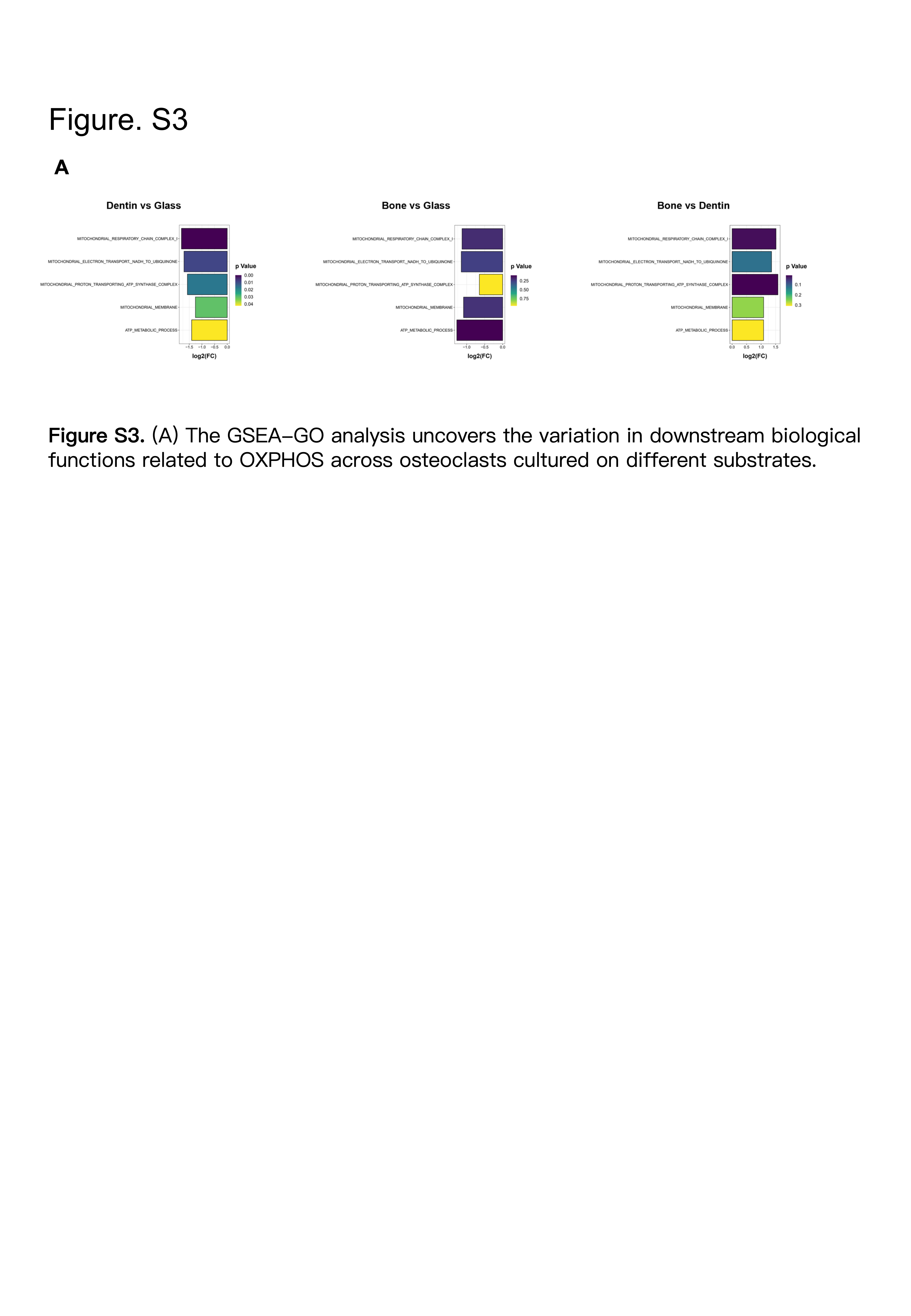

Supplement: Supplementary file 1 [file genes-15-01217-s001.zip › Figure S3.png]
